# Rationale and design of the Edwards SAPIEN-3 periprosthetic leakage evaluation versus Medtronic CoreValve in transfemoral aortic valve implantation (ELECT) trial: A randomised comparison of balloon-expandable versus self-expanding transcatheter aortic valve prostheses

**DOI:** 10.1007/s12471-016-0934-3

**Published:** 2016-12-09

**Authors:** M. Abawi, P. Agostoni, N. H. M. Kooistra, M. Samim, F. Nijhoff, M. Voskuil, H. Nathoe, P. A. Doevendans, S. A. Chamuleau, K. Urgel, J. Hendrikse, T. Leiner, A. C. Abrahams, B. van der Worp, P. R. Stella

**Affiliations:** 1 Department of Cardiology, University Medical Centre Utrecht, Utrecht, The Netherlands; 2 Department of Cardiology, St. Antonius Hospital, Nieuwegein, The Netherlands; 3 Department of Radiology, University Medical Centre Utrecht, Utrecht, The Netherlands; 4 Department of Nephrology and Hypertension, University Medical Centre Utrecht, Utrecht, The Netherlands; 5 Department of Neurology, University Medical Centre Utrecht, Utrecht, The Netherlands

**Keywords:** Transcatheter aortic valve implantation, Periprosthetic aortic regurgitation, SAPIEN-3, CoreValve

## Abstract

**Background and objectives:**

Periprosthetic aortic regurgitation (PPR) after transcatheter aortic valve implantation (TAVI) remains an important issue associated with impaired long-term outcomes. The current randomised study aims to evaluate potential differences between the balloon-expandable Edwards SAPIEN-3 and the self-expanding Medtronic CoreValve system with the main focus on post-TAVI PPR by means of novel imaging endpoints, and an additional focus on other clinical endpoints.

**Endpoints:**

The primary endpoint of this study is quantitative assessment of the severity of post-procedural PPR using cardiac magnetic resonance imaging. Several other novel imaging modalities (X-ray contrast angiography, echocardiography) are used as secondary imaging modalities for the assessment of PPR following TAVI. Secondary objectives of the study include clinical outcomes such as cerebral and kidney injury related to TAVI, and quality of life.

**Methods and design:**

The ELECT study is a single-centre, prospective, two-armed randomised controlled trial. For the purpose of this study, 108 consecutive adult patients suitable for transfemoral TAVI will be randomly allocated to receive the SAPIEN-3 (*n* = 54) or the CoreValve system (*n* = 54).

**Discussion:**

The ELECT trial is the first randomised controlled trial to quantitatively compare the extent of post-TAVI PPR between the SAPIEN-3 and CoreValve. Furthermore, it will evaluate potential differences between the two prostheses with regard to mid-term clinical outcome and quality of life.

## Background

Transcatheter aortic valve implantation (TAVI) is a valid treatment strategy for patients with severe symptomatic aortic stenosis who are regarded as being at high risk [1, 2] or as unable to undergo open-heart surgery [3]. Two transcatheter heart valves (THVs) based on different technical concepts have been developed and are widely used. These are the balloon-expandable SAPIEN THV (Edwards Lifesciences, Irvine, CA, USA), and the self-expanding CoreValve THV (CV-THV) (Medtronic, Minneapolis, MN, USA). Both THVs have shown excellent clinical results, but each has specific features, advantages and disadvantages [2, 4, 5].

Currently available evidence suggests that TAVI is feasible and provides long-term haemodynamic and clinical improvements, but questions remain concerning the safety and durability of this technique. Several important complications of TAVI have to be addressed in order to warrant the wider use of this procedure [6, 7]. Significant concerns have been raised about the high incidence of post-procedural periprosthetic aortic regurgitation (PPR) [1, 2] which is associated with increased mortality [8–11]. Other important procedural-related complications are cerebrovascular events [12], cardiac conduction disorders [13] and acute kidney injury [14, 15].

It has been estimated that between 41–100% of patients have some degree of PPR following TAVI (mild >45%; moderately severe 2–12%) [10, 16]. Even mild PPR is associated with 10–15% higher mortality after two years in comparison with patients with either no or only a trace of PPR, as shown in the PARTNER cohort A [4]. The extent and severity of native valve and left ventricular outflow tract (LVOT) calcification, undersizing of the valve prosthesis relative to the dimensions of the aortic annulus, suboptimal placement of the prosthesis and incomplete apposition of the stent frame owing to calcification in the device landing zone are all known mechanisms of PPR following TAVI [17]. However, the impact of prosthesis type (S3-THV or CV-THV) on the risk of post-TAVI PPR is less clear.

A few recent reports [17, 18], one of which was the only randomised trial to compare SAPIEN XT-THV (SXT-THV) (Edwards Lifesciences, Irvine, CA, USA) with CV-THV (CHOICE study), have suggested differences in the haemodynamic performance of both THVs where the use of CV-THV was associated with a higher rate of residual PPR. However, the operator’s familiarity with the device and subjective measurement of PPR using unidimensional X‑ray angiography images or two-dimensional (2D) echocardiography may bias the results. Hence, an adequately powered randomised study using quantitative assessment techniques may clarify the difference in severity of PPR between SXT-THV and CV-THV more reliably.

Although 2D echocardiography is currently considered the reference standard for evaluation and grading of aortic regurgitation, PPR is difficult to detect and quantify using conventional 2D echocardiography. In PPR, eccentric jets may become entrained along the left ventricular wall, which tends to alter their appearance and hence the perception of PPR severity [19]. Moreover, the possible presence of multiple jets originating from different periprosthetic locations makes their cumulative impact on the overall importance of PPR difficult to judge. Furthermore, in obese patients and patients with chronic obstructive pulmonary disease, the acoustic window at transthoracic echocardiography may be inadequate for comprehensive examination of the valves. Finally, this evaluation is also subject to inter- and intra-observer variability. For these reasons, other diagnostic modalities such as three-dimensional transoesophageal echocardiography (3D-TEE) [20, 21], phase contrast magnetic resonance imaging (MRI) [22], and X‑ray contrast aortography [23] have been proposed for quantification of PPR after TAVI. These modalities can be used to measure the severity of aortic regurgitation by quantifying regurgitant volume and regurgitant fraction, thus minimising observer dependency.

Recently, the SAPIEN-3 THV (S3-THV) (Edwards Lifesciences, Irvine, CA, USA) was introduced [24] which, besides a differently designed stent frame, has an additional outer skirt in order to minimise post-TAVI PPR [5, 25]. However, whether this new feature will be effective in preventing post-TAVI PPR in daily practice remains to be established as well as the direct comparison versus the CV-THV. We designed a clinical trial for the randomised comparison of S3-THV and CV-THV in a routine TAVI population focusing primarily on post-TAVI PPR measured by different innovative imaging modalities, which allow a more accurate and observer-independent quantification of the PPR.

## Methods and design

The ELECT study is a single-centre, prospective, two-armed randomised controlled trial. Consecutive adult males or females, judged eligible for transfemoral TAVI by the local multidisciplinary heart team, and meeting the inclusion criteria will be approached for inclusion in this study. Device size selection will be based on charts provided by the manufacturer and on the pre-procedural multislice computed tomography (MSCT) annular perimeter. The ELECT trial has been designed in accordance with the ethical principles of the Declaration of Helsinki. The Ethics Committee of the University Medical Center Utrecht has given its full approval for the study and patients who agree to participate will be asked for written informed consent.

### Inclusion criteria

In order to be eligible to participate in this study, subjects must meet all of the following criteria: The patient must be ≥18 years of age and diagnosed with severe symptomatic aortic stenosis, judged inoperable or at high surgical risk (EuroSCORE >15% or other criteria that make surgery high risk by a consensus among cardiologists and cardiac surgeons in the heart team) and deemed eligible for transfemoral TAVI. The diameter of the aortic annulus diameter should be ≥18 and ≤28 mm assessed with MSCT. There should be no contraindications to study requirements such as MRI or TEE.

### Exclusion criteria

Patients unable or unwilling to give informed consent are excluded from this study, as are patients whose aortic annulus diameter does not meet the inclusion criteria.

### Randomisation and interventions

**Fig. 1:**
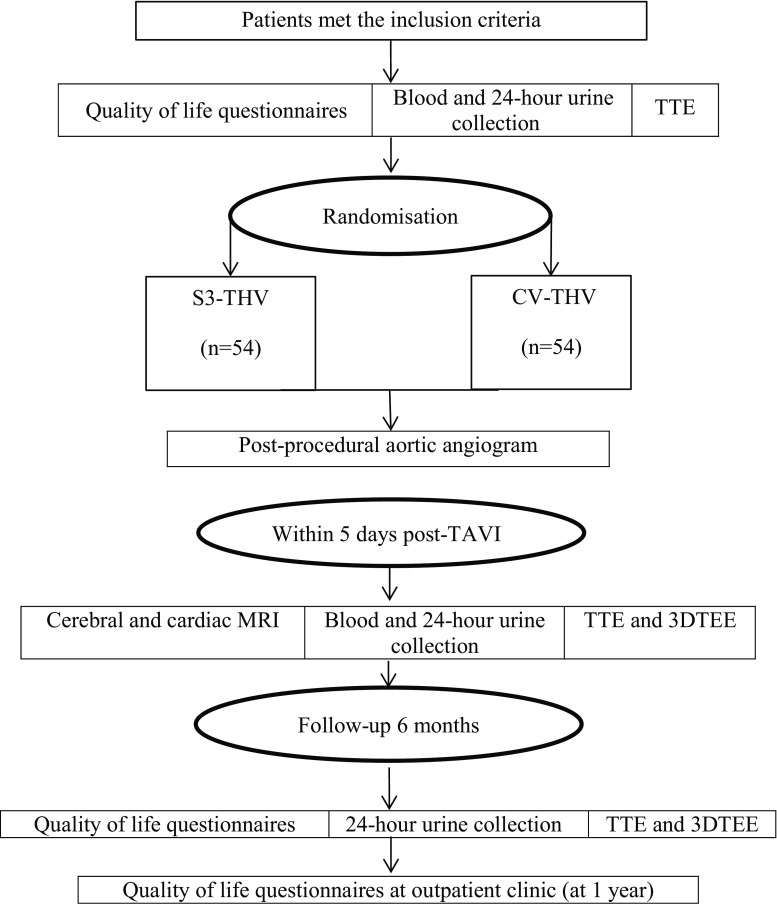
Flowchart of the study

Patients who meet all the inclusion criteria and none of the exclusion criteria will be randomised in consecutive order. After successful puncture of the femoral artery chosen as entry site for TAVI, subjects will be randomised in a 1:1 fashion using sealed envelopes, to receive either an S3-THV or a CV-THV (Fig. 1).

Both prostheses (S3-THV and CV-THV) have received the CE mark of approval for the treatment of severe aortic stenosis. Determination of prosthesis size is based on the aortic annulus diameter, using pre-procedural contrast-enhanced MSCT. The S3-THV is provided in three sizes: 23 mm, 26 mm and 29 mm (for native annulus diameters 18–22 mm, 21–25 mm, and 24–28 mm, respectively). The self-expanding CV-THV is available in four sizes: 23 mm, 26 mm, 29 mm and 31 mm (for native aortic annulus diameters 18–20 mm, 20–23 mm, 23–27 mm, and 27–28 mm, respectively). In order to be able to randomise patients to receive S3-THV or CV-THV, only patients with an annular diameter range between ≥18 and ≤28 mm will be included in this study.

### TAVI procedure

All procedures will be performed by a highly experienced team with extensive knowledge of both devices. Details of the implantation technique for both S3-THVand CV-THV have been previously reported [10, 24, 26, 27]. The procedure is routinely performed under conscious sedation with local anaesthesia. Fluoroscopy and intracardiac echocardiography are used for procedural guidance [28]. Balloon pre-dilatation is performed in all cases in accordance with our routine. Valve deployment is routinely done under rapid pacing (S3-THV 180 beats/min) or stabilising pacing (CV-THV 120 beats/min). Patients are monitored for at least 72 h and discharged on a regimen of lifelong low-dose aspirin (80–100 mg per day), or an oral anticoagulant (if there is a clinical indication for this), and 3 months of clopidogrel (75 mg per day).

### Endpoint assessment

Follow-up assessments for the measurements of the primary and secondary endpoints will be performed at specified time points.

### Primary endpoint

The primary endpoint of this study is quantitative assessment of the severity of post-procedural PPR using CMR. At day 4 (+1) after TAVI, patients without contraindications for MRI will undergo cardiac phase contrast MRI for quantitative grading of PPR according to the standard grading criteria.

### Secondary endpoints

The secondary endpoints of this study include assessment of PPR using other novel imaging modalities including X‑ray contrast angiography, and echocardiography (TTE and TEE). Furthermore, all clinical endpoints as defined by VARC-2 [29] and the quality of life questionnaires will be collected.

**Fig. 2:**
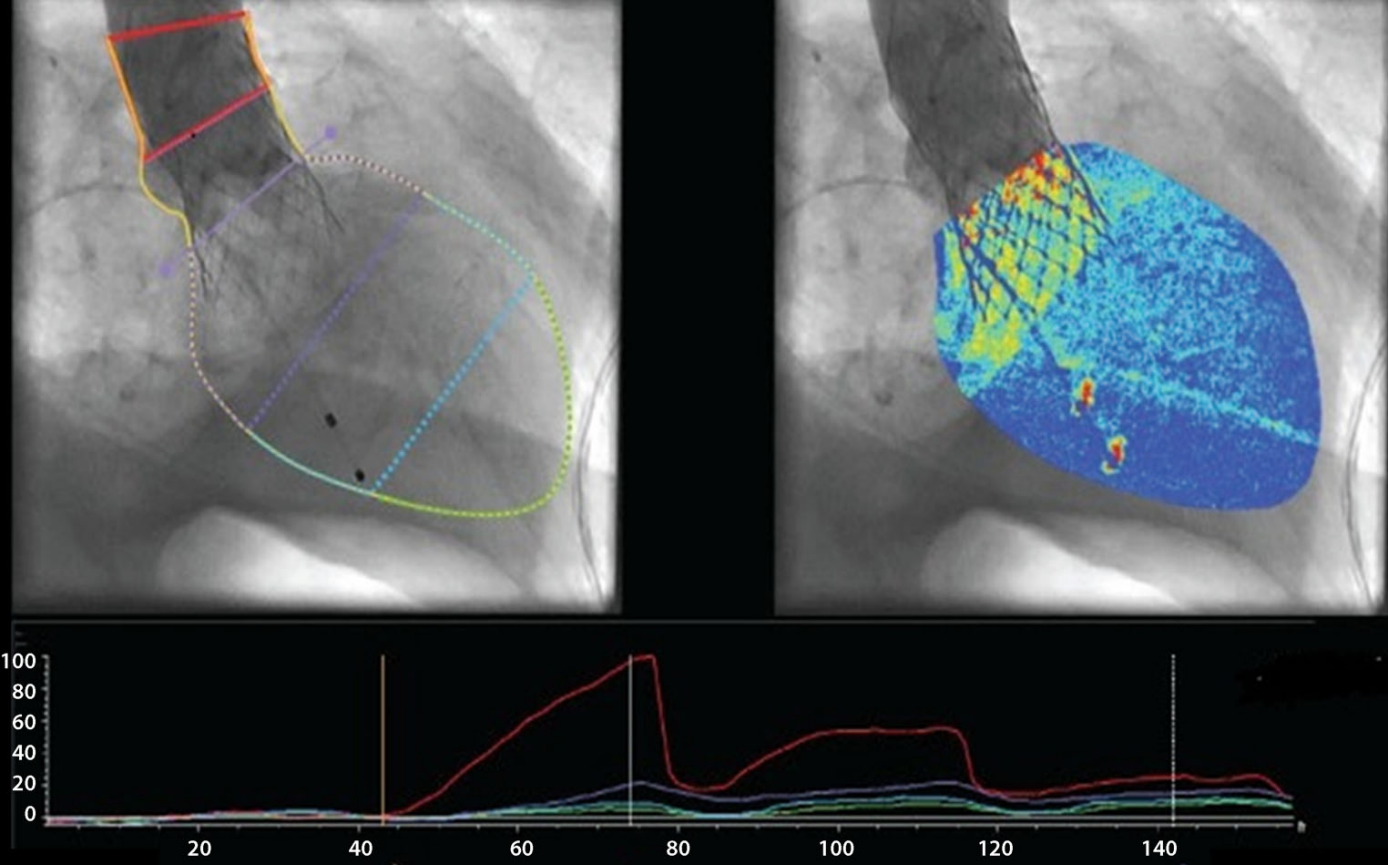
X-ray contrast densitometry with the special software (CAAS A‑Valve) after transcatheter aortic valve implantation

Contrast X‑ray angiography is performed at the end of each TAVI procedure in order to measure the degree of post-implantation PPR by contrast densitometry (CAAS A‑valve quantitative regurgitation analysis; Pie Medical Imaging, Maastricht, the Netherlands) (Fig. 2). Technical details of this approach have been reported previously [23].

Echocardiography, including TTE and TEE, is performed within five days following TAVI. The use of the Doppler technique provides accurate information on the number and severity of paravalvular jets. In addition, 3D TEE is used to analyse the complete morphology of the PPR colour flow stream in the region of its origin. Consequently, views from any level can be obtained, and the direction and extension of PPR jets can be assessed. New dedicated analysis software (Personal Space Technologies BV, the Netherlands) designed for the visualisation and analysis of 3D volumetric echocardiography data will be used for the quantitative grading of PPR severity in this study.

**Fig. 3:**
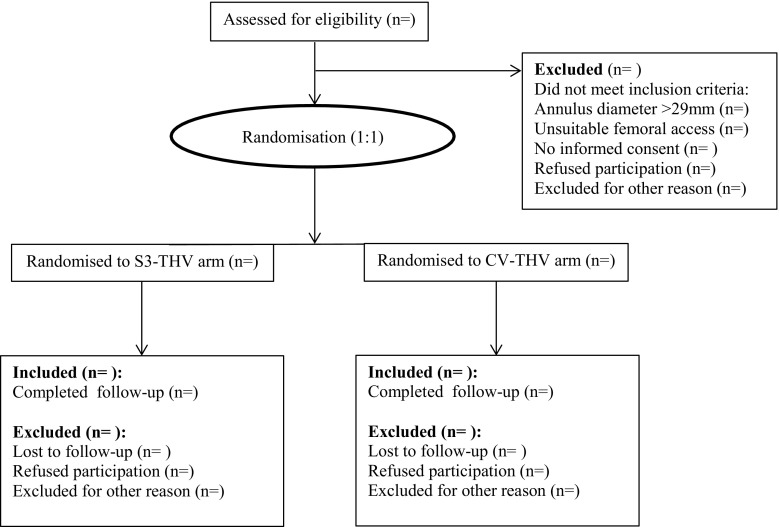
Planned flow diagram during ELECT trial

Patients will undergo cerebral MRI including a diffusion-weighted imaging sequence on day 4 (+1) after TAVI, for detection of new cerebral ischaemic injury [30]. In order to detect kidney injury related to TAVI, serum and urine samples (including 24-hour urine) will be collected. Acute kidney injury will be assessed within 5 days after TAVI and compared with baseline. Irreversible kidney injury (VARC-2) will be assessed at 6‑month follow-up. In order to investigate possible mid-term changes in post-TAVI PPR, valvular function will be evaluated at 6 months using TTE and 3D TEE as described above. Finally, validated quality of life questionnaires (KCC-Q, EuroQol EQ-5D and SF-36) will be used to measure health-related quality of life at one year follow-up and compared with baseline (Fig. 3).

## Statistical analysis

Descriptive statistics will be used to ascertain any imbalance between the arms at baseline. The extent of missing data will be reported and baseline factors will be compared for completers and non-completers to assess the extent of any bias that may result.

Continuous variables are presented as means ± standard deviation or medians [interquartile range], as considered appropriate, and categorical variables as counts and percentages. Continuous variables will be compared using the Student *t* test or Mann-Whitney test, depending on data distribution. Categorical variables will be compared using chi-square or Fisher’s exact test, as considered appropriate. For the analysis of the difference in PPR between each of the prostheses, log transformation will be performed on the regurgitation volume data before performing a *t* test, as these data are expected to be right-skewed. The follow-up analysis for changes in the endpoints in the time within the arms will be performed using the paired *t* test and the repeated measurements analysis of variance. A two-tailed *p*-value of less than 0.05 is regarded as statistically significant. All analyses will be performed according to the intention-to-treat principle. Data will be analysed using IBM SPSS Statistics software version 20 (IBM Corp., Armonk, NY, USA), and R version 2.12.0 (http://www.r-project.org).

### Sample size calculation

A sample size calculation for the primary endpoint post-TAVI PPR has been performed. A literature search on the difference in the extent of PPR after TAVI between the S3-THV and CV-THV prostheses has been done. No studies were found that included a quantitative PPR comparison between these two prostheses. We used data from 25 patients treated with TAVI at our centre for a sample size calculation. PPR quantification in these patients was performed using cardiac MRI. Because the distribution of these volume data was skewed to the right, we performed a log transformation on the original data to improve approximations to normality (original volumes presented as ml). The log transformed values were as follows: an overall mean regurgitant volume of 0.48, an ‘overall’ median regurgitant volume of 0.48, an overall standard deviation (SD) of 0.53, and a mean regurgitant volume of 0.32 for S3-THV and a mean regurgitant volume of 0.63 for CV-THV. CMR grades of PPR will be defined according to regurgitation fraction using similar reference cut-point values according to the standard grading criteria. As even mild PPR has been shown to be associated with long-term mortality, any significant difference in post-TAVI PPR volume between S3-THV and CV-THV is considered clinically important. The sample size calculation, including the above-mentioned log transformed values for mean regurgitant volumes and standard deviations, yielded a sample size of 49 patients in each arm to show superiority or equivalence over S3-THV and CV-THV with a power of 80%. As we took an expected dropout rate of 10% into account, this yielded a total sample size of 54 patients in each arm.

## Discussion

The ELECT trial is the first randomised controlled trial to quantitatively compare the magnitude of post-procedural PPR between the S3-THV prosthesis and CV-THV. Other important objectives of this trial include assessment of the value of angiography, transthoracic and (3D) transoesophageal echocardiography and cardiac MRI for the measurement of post-TAVI PPR severity.

Regardless of the prosthesis type, post-procedural PPR is a common complication after TAVI associated with increased mortality [17]. A meta-analysis [25] including 45 studies reported an overall incidence of moderate or severe PPR of 11.7% within 30 days after TAVI. The balloon-expandable S3-THV [24] and self-expanding CV-THV [31] are two transcatheter heart valves that are in widespread use worldwide. Few previous studies comparing the haemodynamic performance of these two types of prosthesis have suggested a higher incidence of moderate or severe post-TAVI PPR accompanying the implantation of CV-THV [1, 25].

An important aspect of CV-THV that might increase the risk of post-TAVI PPR (and thus explain the aforementioned data) is the intrinsic radial strength of its nitinol frame, which may not be sufficient for complete apposition of the prosthesis to the native annulus. This incomplete apposition might create periprosthetic gaps, especially in the presence of calcification along the aortic wall. Furthermore, an extreme angulation between the left ventricular outflow tract and the ascending aorta (also called the horizontal aorta) may reduce the ability of the CV-THV prosthesis to seal the paravalvular space [25]. Therefore, oversizing and balloon post-dilatation are more important in CV-THV implantation than in S3-THV implantation. In addition, owing to the non-cylindrical shape of the CV-THV system, its effective area inside the aortic annulus depends on the depth of the prosthesis in the left ventricular outflow tract. Therefore, in the CV-THV the sealing of the paravalvular space by the prosthesis also depends on the depth of its implantation. However, besides the factors mentioned above, which make CV-THV susceptible to post-TAVI PPR, we also need to keep in mind that the self-expanding nitinol frame has the potential to further expand over time. Therefore, the apposition of the prosthesis frame to the aortic annulus has the potential to improve gradually, decreasing the extent of post-TAVI PPR with passage of time.

Interestingly, the recently published results of the CoreValve US Pivotal Trial [32] showed that the incidence of any PPR after the implantation of CV-THV decreases over time: 41.1% at discharge and 31.9% at one year. The latter was also accompanied by a reduction in the incidence of more-than-mild aortic regurgitation during follow-up, at 13.8% at discharge, 10.1% at 6 months, and 6.4% at one year. This suggests that measurement of post-TAVI PPR in the CV-THV during the few first days after TAVI will probably overestimate its long-term severity. In the ELECT study, the severity of PPR is also measured at 6‑month follow-up using transthoracic and 3D transoesophageal echocardiography, which allows for a more reliable comparison between the two prostheses.

One previously published meta-analysis [25] showed a higher risk of moderate or severe post-procedural PPR after CV-THV implantation (16%) as compared with E﻿d﻿w﻿a﻿r﻿d﻿s SAPIEN implantation (9.1%, *p* = 0.005). The CHOICE study [18], which is the only randomised head-to-head trial to date that has compared the balloon-expandable SXT-THV with the CV-THV system, reported a significant difference in the frequency of any degree of PPR (38% in SXT group versus 65% in the CV group, *p* < 0.001) and more-than-mild PPR (4.1% in SXT group versus 18.3% in the CV group, *p* < 0.001 by angiography) favouring the balloon-expandable valve. However, the extent of the PPR in the CHOICE trial was measured using aortic root angiography immediately after prosthesis implantation, which is an important limitation of this clinical trial.

On the other hand, the PRAGMATIC [33] study reported a very low and comparable incidence of more-than-mild PPR with both Edwards SAPIEN and CV-THV (1.8% and 2.0%, respectively) in a large multicentre propensity score-matched study. Recently, another study among a selected group of patients receiving S3-THV or CV-THV reported a significantly lower rate of mild or severe PPR, lower need for permanent pacemaker implantation, and higher rate of device success for the S3-THV compared with CV-THV. However, this study was limited by its design and small sample size [34].

Obviously, there is a discrepancy in the incidence of PPR across different studies that is most probably related to the challenges in identification and quantification of post-TAVI PPR. Also, the VARC-2 document [29] did not propose new diagnostic criteria for adequate assessment of post-TAVI PPR. The lack of a standardised and validated method for evaluation of post-TAVI PPR is a major limitation in comparing echocardiographic PPR analysis performed in different studies and meta-analyses. Aortic regurgitation after TAVI usually consists of multiple eccentric jets that are non-parallel and irregular in shape [21, 35].

Eccentric jets are frequently entrained along the left ventricular wall with fanning of jets as they regurgitate. Therefore, the eccentric aspects of post-TAVI PPR make the assessment of its severity challenging. Also acoustic shadowing from the calcifications and Doppler attenuation from the prosthesis can obscure regurgitant jets and thus result in underestimation of their severity [35].

On the other hand, assessment of PPR severity using aortic root angiography relies on subjective assessment of unidimensional images, and can be affected by inter-observer and intra-observer variability.

In the ELECT trial, the severity of PPR is measured quantitatively using several different modalities, from dedicated software for angiography [23] directly post-TAVI implantation to either transthoracic and transoesophageal echocardiography (with 3D reconstruction) [20, 21] to cardiac MRI including phase contrast sequences [22]. The main focus of the ELECT trial will be the comparison of the different imaging modalities in assessing PPR, using CMR as a primary endpoint. This comparison is an important aspect of the present trial as to date there is no validated tool for reliable measurement of post-TAVI PPR.
